# A high content, small molecule screen identifies candidate molecular pathways that regulate rod photoreceptor outer segment renewal

**DOI:** 10.1038/s41598-018-32336-y

**Published:** 2018-09-18

**Authors:** Leah J. Campbell, Megan C. West, Abbie M. Jensen

**Affiliations:** 1 Biology Department, University of Massachusetts, Amherst, MA 01003 USA; 2 Molecular and Cellular Biology Graduate Program, University of Massachusetts, Amherst, MA 01003 USA; 3 Present Address: Department of Biological Sciences, University of Notre Dame, Notre Dame, IN 46556 USA

## Abstract

The outer segment of the vertebrate rod photoreceptor is a highly modified cilium composed of many discrete membranous discs that are filled with the protein machinery necessary for phototransduction. The unique outer segment structure is renewed daily with growth at the base of the outer segment where new discs are formed and shedding at the distal end where old discs are phagocytized by the retinal pigment epithelium. In order to understand how outer segment renewal is regulated to maintain outer segment length and function, we used a small molecule screening approach with the transgenic *(hsp70:HA-mCherry*^*TM*^) zebrafish, which expresses a genetically-encoded marker of outer segment renewal. We identified compounds with known bioactivity that affect five content areas: outer segment growth, outer segment shedding, clearance of shed outer segment tips, Rhodopsin mislocalization, and differentiation at the ciliary marginal zone. Signaling pathways that are targeted by the identified compounds include cyclooxygenase in outer segment growth, γ-Secretase in outer segment shedding, and mTor in RPE phagocytosis. The data generated by this screen provides a foundation for further investigation of the signaling pathways that regulate photoreceptor outer segment renewal.

## Introduction

Vertebrate photoreceptors are specialized light-sensing neurons with unique morphology that is essential for function. The compartmentalized structure includes a highly modified cilium called the outer segment, which contains densely stacked membranous discs. These discs are packed with the phototransduction machinery that absorbs and converts light into the membrane potential change that alters neurotransmitter release. Blinding diseases such as retinitis pigmentosa and macular degeneration are characterized by degeneration and loss of photoreceptors^1,2^. Therefore, a better understanding of the cellular maintenance of the photoreceptor outer segment may provide guidance for the design and optimization of treatments to prevent vision loss and restore or prolong vision.

The rod photoreceptor outer segment (ROS) contains on the order of 1,000 discrete discs that are stacked perpendicularly to the ciliary axoneme^3^. In order to supply the ROS with fresh membrane and protein, the ROS undergoes a unique process of continuous renewal. Using autoradiography to detect pulse-labeled H^3^-proteins, it was observed that new protein-packed discs are regularly added to the base of the ROS^4,5^. Recent studies give strong evidence to the evagination model of disc formation where new discs develop as evaginations of the ciliary plasma membrane and successive evaginations fuse to form the discrete discs^6–8^. The tips of outer segments, which contain the oldest discs and associated proteins, are recognized, phagocytosed, and digested diurnally by the neighboring retinal pigment epithelium (RPE)^9,10^.

ROS renewal is regulated, in part, by light as demonstrated by reports that exposure to light inhibits delivery of Rhodopsin to the ROS^11^ and that the shedding event is initiated by illumination^12,13^. In addition, phosphodiesterase inhibitors can mimic the dark state to prevent ROS shedding^14^. Beyond this, our understanding of the mechanisms that regulate renewal are limited. Stress from insults to the system, such as mutations in the protein trafficking or ciliary trafficking machinery that disrupt the delivery of molecules to the OS, results in photoreceptor degeneration^15^. Disruption of RPE phagocytic function caused by mutations in the Mertk receptor in the RPE^16–18^ and mutations in the Mertk ligands, Gas6 and Protein S^19^, also result in degeneration. Understanding the regulation of growth and shedding at a molecular level will provide better insight of how renewal is balanced for healthy maintenance of the outer segment.

A major obstacle in the progress towards identifying the mechanisms regulating ROS renewal has been the inability to easily and quantitatively measure growth and shedding kinetics. To accelerate progress, we created a transgenic zebrafish, *Tg(hsp70:HA-mCherry*^*TM*^), that allows us to quickly and quantitatively measure ROS growth and shedding^20^. Given the high amenability of zebrafish to chemical screens^21^, we screened a library of compounds with known bioactivity using *Tg(hsp70:HA-mCherry*^*TM*^) fish to identify modulators of ROS renewal. In addition to the primary objective of identifying molecular pathways involved in ROS renewal, we also examined Rhodopsin localization, clearance of shed ROS material by the RPE, and the addition of new rod photoreceptors from the ciliary marginal zone (CMZ) of the retina.

## Results

### High content screen of 1351 bioactive compounds on ROS renewal

ROS renewal occurs only in the intact retina, and efforts to recapitulate the process by culturing rods have been unsuccessful^22^. The zebrafish offers several unique advantages as an *in vivo* model for ROS renewal studies, including the amenability for compound screening. The zebrafish retina develops rapidly with large numbers of ROS present as early as 5 days post fertilization (dpf)^23^. It is easy to generate the large numbers of individual animals needed for a bioactive compound screen, and compounds can be delivered by bathing the zebrafish in small volumes of water that require small amounts of compound. Finally, the *Tg(hsp70:HA-mCherry*^*TM*^) line provides a quicker method for measuring ROS renewal kinetics than the time-consuming and cumbersome autoradiography, which relies on detection of radioactively-labeled proteins and has been rarely used since the 1970s^4,5,20^.

**Figure 1:**
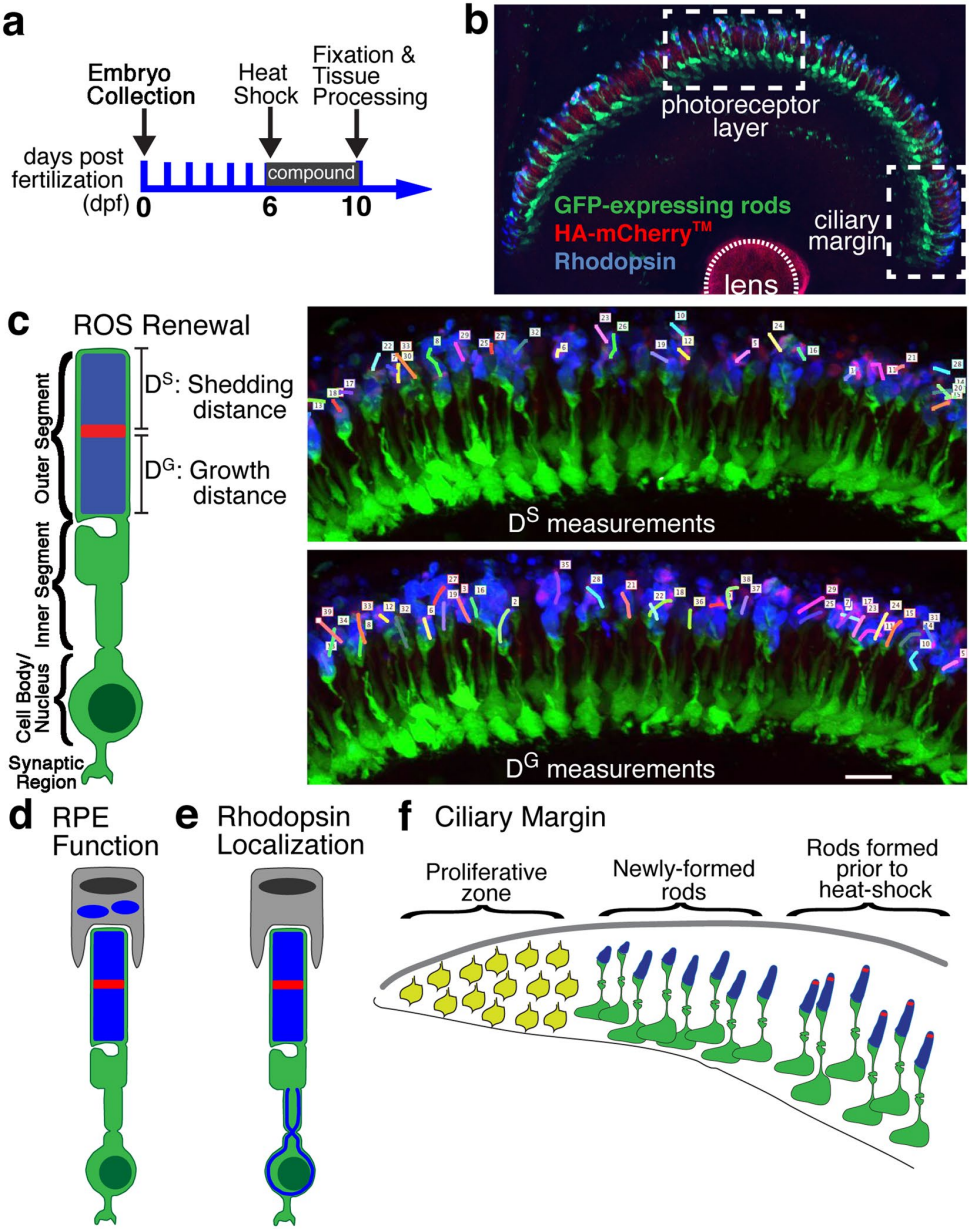
High-content screening of 1351 bioactive small molecules for ROS renewal. (**a**) Work-flow for high-content, image-based screen. *Tg(Xla*.*rho:EGFP); Tg(hsp70:HA-mCherry*^*TM*^*); alb*^−/−^ fish were heat shocked at 6 dpf to induce expression of HA-mCherry^TM^ followed by rearing in 20 μM compound until 10 dpf when tissues were fixed and processed for immunolabeling and confocal microscopy. (**b**) Confocal z-stack images were acquired of the GFP (green) and HA-mCherry^TM^ stripe (red)-expressing rods labeled with anti-Rhodopsin antibody (blue) in two regions of interest: the photoreceptor layer and ciliary margin (white-hatched boxes). (**c**) ROS renewal was measured within the three-dimensional z-stacks with the growth distance (D^G^) representing the distance from the base of the ROS to the mCherry^TM^ stripe and the shedding distance (D^S^) representing the distance from the mCherry^TM^ stripe to the tip of the outer segment. Scale bar is 10 μm. (**d**,**e**) Qualitative changes in the photoreceptor layer were documented, including the (**d**) accumulation of phagosomes and (**e**) Rhodopsin mislocalization. (**f**) Qualitative changes in the ciliary margin were documented, such as the size of the proliferative zone and addition of new rods during compound treatment, which can be identified by lack of the mCherry^TM^ stripe.

To identify potential pathways that regulate ROS renewal, we tested 1351 compounds with known bioactivity. Figure 1 describes the screening approach. At 6 dpf, *Tg(hsp70:HA-mCherry*^*TM*^)*; Tg(Xla*.*rho:EGFP*)*; alb*^*−/−*^ fish were heat shocked to induce expression of the HA-mCherry^TM^ marker of ROS renewal (mCherry^TM^ stripe) and then immediately transferred to tissue culture wells containing 20 μM of compound or 0.2% DMSO (vehicle control). At 10 dpf (4 days post heat shock), fish were fixed and processed for immunofluorescence (Fig. 1a). Antibodies against GFP and the HA-epitope in the mCherry^TM^ stripe were used to extend the lifetime and enhance the fluorescence of these proteins; antibody against Rhodopsin was used to label the ROS and reveal possible effects on Rhodopsin localization. Confocal z-stacks of the photoreceptor layer and of the peripheral rim of the retina, which includes the CMZ, were collected for image analysis (Fig. 1b). The primary goal of the screen was to identify potential molecular pathways that regulate ROS renewal. ROS growth and shedding were measured within the 3-dimensional confocal z-stacks as the distance from the base of the ROS to the mCherry^TM^ stripe (D^G^) and the distance from the mCherry^TM^ stripe to the tip of the ROS (D^S^), respectively (Fig. 1c). To expand the utility of the screen, we also qualitatively examined the photoreceptor layer and CMZ for 3 other content areas: the accumulation of phagosomes as an indication of disrupted function of the retinal pigmented epithelium (RPE) to phagocytose and/or digest shed ROS material (Fig. 1d), Rhodopsin mislocalization as an indication of disrupted trafficking (Fig. 1e), and shape of the peripheral rim and CMZ for disrupted addition of new rods (Fig. 1f). See Materials and Methods for details on the screen design, data collection, and analysis. The complete list of the screened compounds, of which approximately 30% were lethal at the tested concentration, and resulting data are provided in Supplementary Table S1.

### ROS growth is disrupted by bioactive compound treatment

**Figure 2:**
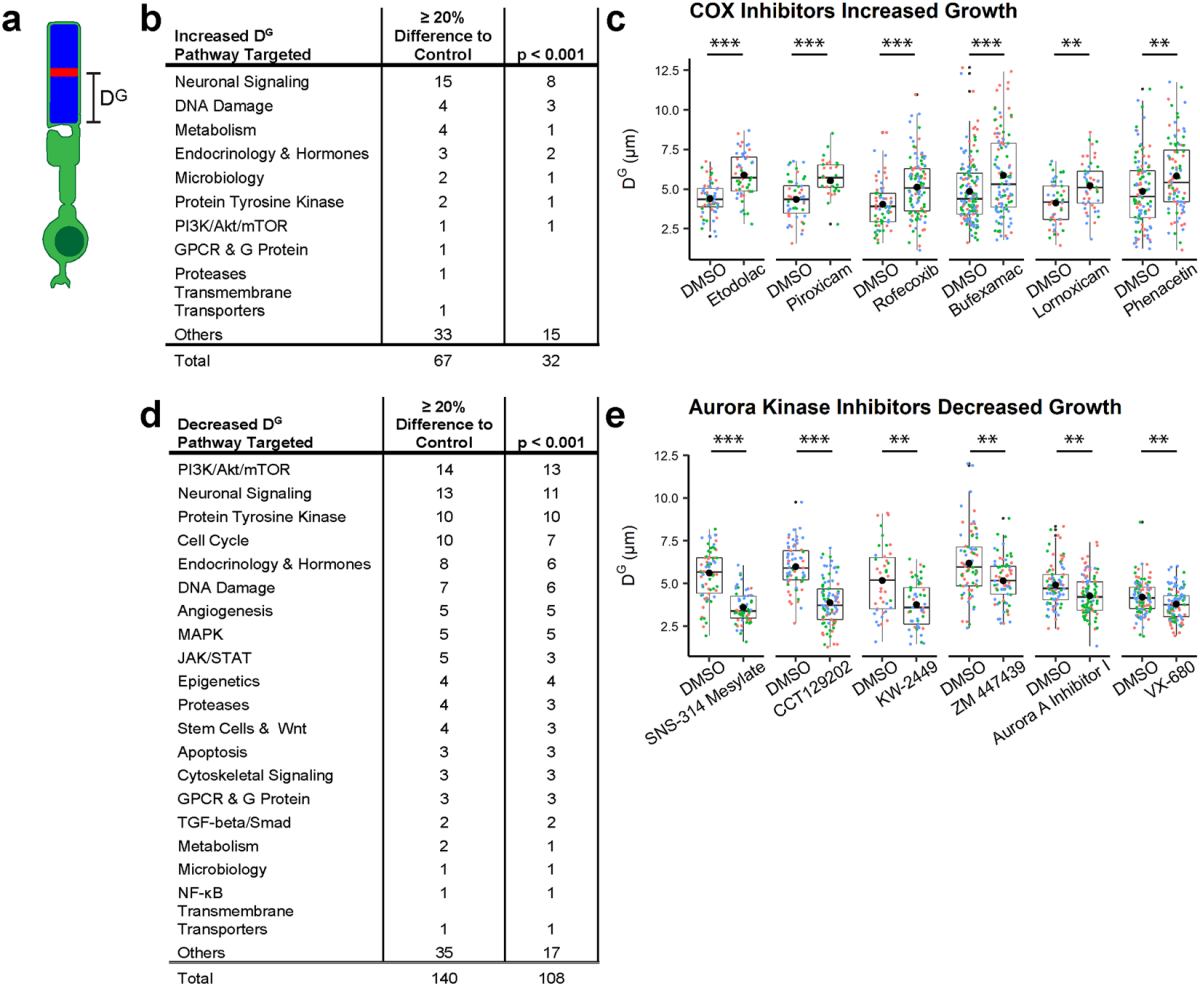
Cyclooxygenase and Aurora Kinase are among targets identified for ROS growth regulation. (**a**) D^G^, measured as the distance from the base of the ROS to the mCherry^TM^ stripe, represents the ROS growth that occurred during compound treatment. (**b**) Signaling pathways targeted by compounds that increased D^G^. (**c**) COX inhibitors increased D^G^ as compared to DMSO control (0.2%). (**d**) Signaling pathways targeted by compounds that decreased D^G^. (**e**) Aurora Kinase inhibitors decreased D^G^ as compared to DMSO control (0.2%). Each ROS D^G^ measurement is represented by a small dot of color that represents an individual fish (*n* = 33–149 rods from 3 fish). Lower and upper hinges of box plots correspond to first and third quartiles; middle line corresponds to median; whiskers extend 1.5 * interquartile range above and below the hinges; large single black dot represents the mean; small black dots represent outliers. ***p < 0.001; **p < 0.01 by Dunnett’s comparison.

D^G^ represents the ROS growth that occurred after heat shock and during the 4 days of compound treatment (Fig. 2a). D^G^ can be highly variable between independent experiments. This is attributable to variation between ROS within a retina as well as variation between individual fish from the same compound treatment. The variability is due to any combination of intrinsic and extrinsic variables that affects overall growth and development of the fish, such as egg quality, temperature, water quality, and the availability and quality of food at first-feeding^24^. In order to account for this variability, the ROS measurements from 3 fish treated with the same compound were combined and analyzed with mixed effects modeling to determine mean and standard deviation. Mean D^G^ from compound-treated fish was compared to mean D^G^ from DMSO control-treated fish that were fertilized within the same clutch and processed through the screen concurrently. Comparisons were made using the Dunnett comparison procedure^25^ with the adjusted *p*-values from the mixed effects modeling.

In order to identify compounds of potential interest, we first determined a benchmark for D^G^ by analyzing all of the DMSO control-treated fish that were processed during independent iterations of the screen (n = 213 fish with a total of 5,246 measured ROS). The mean D^G^ for all DMSO controls was 5.01 μm (±0.650 μm) or 1.25 μm per day (Table 1). This D^G^ benchmark of 5.01 μm represented an expected average D^G^ for the DMSO control but was not an appropriate value to compare with individual compound treatments due to the variability discussed above. Instead, the benchmark was used to set a threshold for changes in D^G^. A 1 μm difference in the benchmark D^G^ during the 4 days post heat shock of the screen assay would represent a 20% change. Using this as a biological threshold, we focused initial attention on those compounds that increased D^G^ by at least 20% as compared to the associated DMSO control and with statistical significance of *p* < 0.001. Upon identifying compounds of interest, we examined other compounds with the same target that similarly affected D^G^ although perhaps outside of the biological (≥20%) and statistical (*p* < 0.001) thresholds.

**Table 1:** D^G^ and D^S^ for ROS from all DMSO vehicle control-treated fish in study.

	mean	standard deviation	total number of ROS	total number of retinas
D^G^	5.01 μm	0.650 μm	5246	213
D^S^	3.40 μm	0.735 μm	4223	213

We observed 67 compounds that increased D^G^ by at least 20% as compared to the associated control, and 32 were statistically significant with *p* < 0.001. The signaling pathways most commonly targeted were involved in neuronal signaling, as well as DNA damage, metabolism, and endocrinology/hormones (Fig. 2b). Several cyclooxygenase (COX) inhibitors (categorized as pathway “Neuronal Signaling”) significantly increased D^G^ by at least 20% and with *p* < 0.001 as compared to the associated DMSO control (Fig. 2c), including Etodolac by 34% (*p* = 5.00 × 10^−10^; D^G^_Etodolac_ = 5.88 μm ± 0.165 μm, D^G^_Control_ = 4.40 μm ± 0.153 μm), Piroxicam by 27% (*p* = 1.70 × 10^−4^; D^G^_Piroxicam_ = 5.54 μm ± 0.305 μm, D^G^_Control_ = 4.35 μm ± 0.277 μm), Rofecoxib by 27% (*p* = 2.25 × 10^−4^; D^G^_Rofecoxib_ = 5.12 μm ± 0.149 μm, D^G^_Control_ = 4.05 μm ± 0.196 μm), and Bufexamac by 21% (*p* = 3.93 × 10^−4^; D^G^_Bufexamac_ = 5.86 μm ± 0.193 μm, D^G^_Control_ = 4.85 μm ± 0.165 μm). Two additional COX inhibitors were statistically significant at *p < *0.01 as compared to the associated control: Lornoxicam by 26% (*p* = 0.00101; D^G^_Lornoxicam_ = 5.22 μm ± 0.249 μm, D^G^_Control_ = 4.13 μm ± 0.247 μm) and Phenacetin by 20% (*p* = 0.00406; D^G^_Phenacetin_ = 5.82 μm ± 0.288 μm, D^G^_Control_ = 4.85 μm ± 0.279 μm).

We observed 140 compounds that decreased D^G^ by at least 20% as compared to the associated DMSO control, and 108 were statistically significant with *p* < 0.001. The most common pathways targeted by these compounds were involved in mTOR, neuronal signaling, protein tyrosine kinase, cell cycle, endocrinology and hormone, and DNA damage pathways (Fig. 2d). Aurora Kinase inhibitors (categorized as pathway “Cell Cycle”) SNS-314 Mesylate (D^G^_SNS-314 Mesylate_ = 3.58 μm ± 0.513 μm, D^G^_Control_ = 5.61 μm ± 0.282 μm) and CCT129202 (D^G^_CCT129202_ = 3.87 μm ± 0.180 μm, D^G^_Control_ = 5.99 μm ± 0.200 μm) significantly decreased D^G^ by 36% (*p* < 1.00 × 10^−16^), 35% (*p* < 4.00 × 10^−15^) and 25% (*p* = 3.74 × 10^−4^), respectively, as compared to the associated DMSO control (Fig. 2e). Other Aurora Kinase inhibitors that significantly decreased D^G^ at *p* < 0.01 included KW-2449 by 28% (*p* = 0.00143; D^G^_KW-2449_ = 3.76 μm ± 0.275 μm, D^G^_Control_ = 5.18 μm ± 0.303 μm), ZM447439 by 17% (*p* = 0.00161; D^G^_ZM44739_ = 5.16 μm ± 0.200 μm, D^G^_Control_ = 6.19 μm ± 0.201 μm), Aurora A Inhibitor I by 13% (*p* = 0.00973; D^G^_Aurora A Inhibitor I_ = 4.27 μm ± 0.151 μm, D^G^_Control_ = 4.91 μm ± 0.157 μm), and VX-680 by 10% (*p* = 0.00132; D^G^_VX-680_ = 3.78 μm ± 0.0864 μm, D^G^_Control_ = 4.20 μm ± 0.0830 μm). The subset of data for compounds that increased or decreased D^G^ is included in Supplementary Table S1.

### ROS shedding is disrupted by bioactive compound treatment

**Figure 3:**
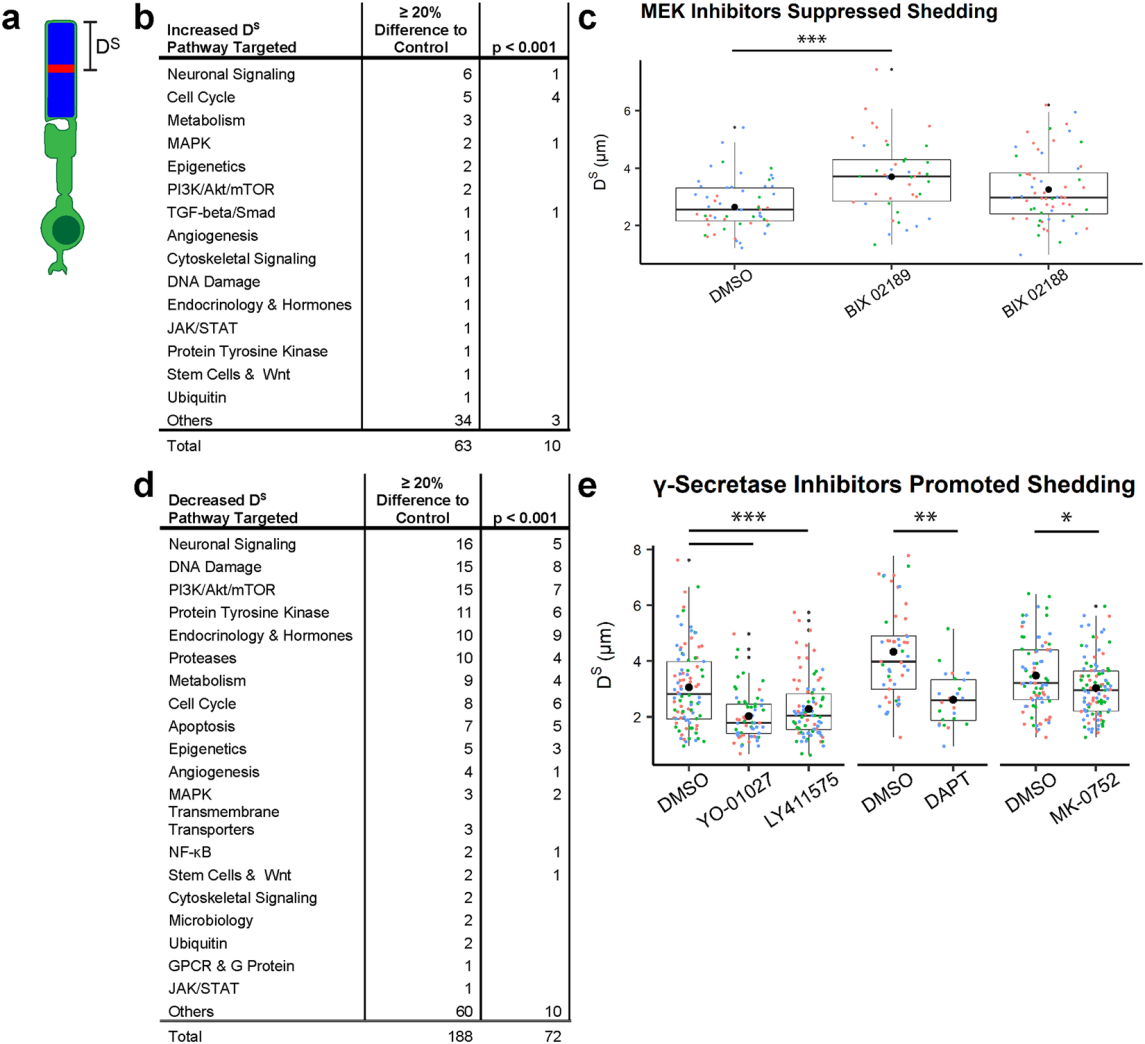
MEK and γ-Secretase are among targets identified for ROS shedding regulation. (**a**) The shedding distance, D^S^, was measured as the distance from the mCherry^TM^ stripe to the tip of the ROS and represents the growth that occurred before heat shock minus the amount shed during compound treatment. (**b**) Signaling pathways targeted by compounds that increased D^S^, and therefore suppressed shedding. (**c**) Two MEK5 inhibitors increased D^S^ as compared to DMSO control (0.2%). Both MEK5 inhibitors were screened during the same week and compared to the same DMSO control. (**d**) Signaling pathways targeted by compounds that decreased D^S^, and therefore promoted shedding. (**e**) γ-Secretase inhibitors increased D^S^ as compared to DMSO control (0.2%). Inhibitors YO-01027 and LY411575 were screened during the same week and compared to the same DMSO control. Each ROS D^S^ measurement is represented by a small dot of color that represents an individual fish (*n* = 24–105 rods from 3 fish). Lower and upper hinges of box plots correspond to first and third quartiles; middle line corresponds to median; whiskers extend 1.5 * interquartile range above and below the hinges; large single black dot represents the mean; small black dots represent outliers. ***p < 0.001; **p < 0.01, *p < 0.05 by Dunnett’s comparison.

D^S^ was determined by measuring the distance from the mCherry^TM^ stripe to the ROS tip. This distance represents the ROS growth that occurred before heat shock minus any shedding during the 4-day compound treatment (Fig. 3a). Again, D^S^ is variable due to subtle experimental conditions and likely inherent biological determinants. Mixed effects modeling was used to analyze D^S^ data, and D^S^ for compound-treated fish was compared (with the Dunnett comparison procedure) to D^S^ for the associated DMSO control-treated fish that were fertilized within the same clutch and processed through the screen concurrently.

In order to identify compounds of potential interest, a benchmark for D^S^ was first determined by analyzing all DMSO control-treated fish from the study (n = 213 fish with a total of 4,223 measured ROS), which gave a mean D^S^ of 3.40 μm ± 0.735 μm (Table 1). Since the D^S^ represents growth minus the amount shed, we had to account for the ROS growth that occurred prior to heat shock. In a previous study, ROS growth in untreated fish that were reared in 14 h light/10 h dark cycle was 1.6 μm per day^14^. This best represented the rearing conditions of fish in this study prior to heat shock at 6 dpf. Therefore, assuming most rod births begin after 3 dpf ^23^, ROS growth prior to heat shock was 4.8 μm (3 days at 1.6 μm per day). Given D^S^ of 3.4 μm, approximately 1.4 μm was shed by DMSO control-treated ROS during the 4 days post heat shock (0.35 μm per day). A 20% increase in the benchmark D^S^ (4.08 μm) would therefore equate to the shedding event suppressed by half (only 0.72 μm shed during the 4 days post heat shock, or 0.18 μm per day). With this benchmark, we focused initial attention on the compounds that increased D^S^ by at least 20% as compared to the associated control and with statistical significance of *p* < 0.001. Other compounds that target the same molecule and similarly affect D^S^, but fall outside of the biological (≥20%) and statistical (*p* < 0.001) thresholds, were identified.

We observed 63 compounds that increased D^S^ by at least 20% as compared to the associated control, and 10 were statistically significant with *p* < 0.001. These compounds targeted pathways involved in neuronal signaling, cell cycle, metabolism, and MAPK signaling, among others (Fig. 3b). BIX 02189, an inhibitor of MEK5 (categorized as pathway “MAPK”), significantly increased D^S^ by 40% (*p* = 1.61 × 10^−4^) as compared to the associated control (D^S^_BIX 02189_ = 3.70 μm ± 0.188 μm, D^S^_Control_ = 2.65 μm ± 0.149 μm; Fig. 3c). In addition, the related MEK5 inhibitor BIX 02188 increased D^S^ by 23% (*p = *0.0538) as compared to the associated control (D^S^_BIX 02188_ = 3.26 μm ± 0.158 μm, D^S^_Control_ = 2.65 μm ± 0.149 μm).

Conversely, a decrease in D^S^ indicates that shedding was increased or stimulated by compound treatment. We observed 188 compounds that decreased D^S^ by at least 20% as compared to control, and 72 were statistically significant with *p* < 0.001. The pathways targeted by these compounds were involved in neuronal signaling, DNA damage, mTOR signaling, protein tyrosine kinase, endocrinology and hormone signaling, and protease pathways (Fig. 3d). YO-01027 (D^S^_YO-01027_ = 2.02 μm ± 0.144 μm, D^S^_Control_ = 3.05 μm ± 0.114 μm) and LY411575 (D^S^_LY411575_ = 2.28 μm ± 0.119 μm, D^S^_Control_ = 3.05 μm ± 0.114 μm) are γ-Secretase inhibitors (categorized as pathway “Proteases”) that significantly decreased D^S^ by 34% (*p* = 3.20 × 10^−7^) and 25% (*p* = 4.57 × 10^−5^), respectively, as compared to the associated control (Fig. 3e). Additional γ-Secretase inhibitors DAPT (D^S^_DAPT_ = 2.61 μm ± 0.399 μm, D^S^_Control_ = 4.33 μm ± 0.283 μm) and MK-0752 (D^S^_MK-0752_ = 3.03 μm ± 0.112 μm, D^S^_Control_ = 3.48 μm ± 0.127 μm) also decreased D^S^ as compared to the associated control by 40% (*p* = 0.00253) and 13% (*p* = 0.0304), respectively. The subset of data for compounds that increased or decreased D^S^ is included in Supplementary Table S1.

### Bioactive compounds inhibit RPE clearance of shed ROS material

**Figure 4:**
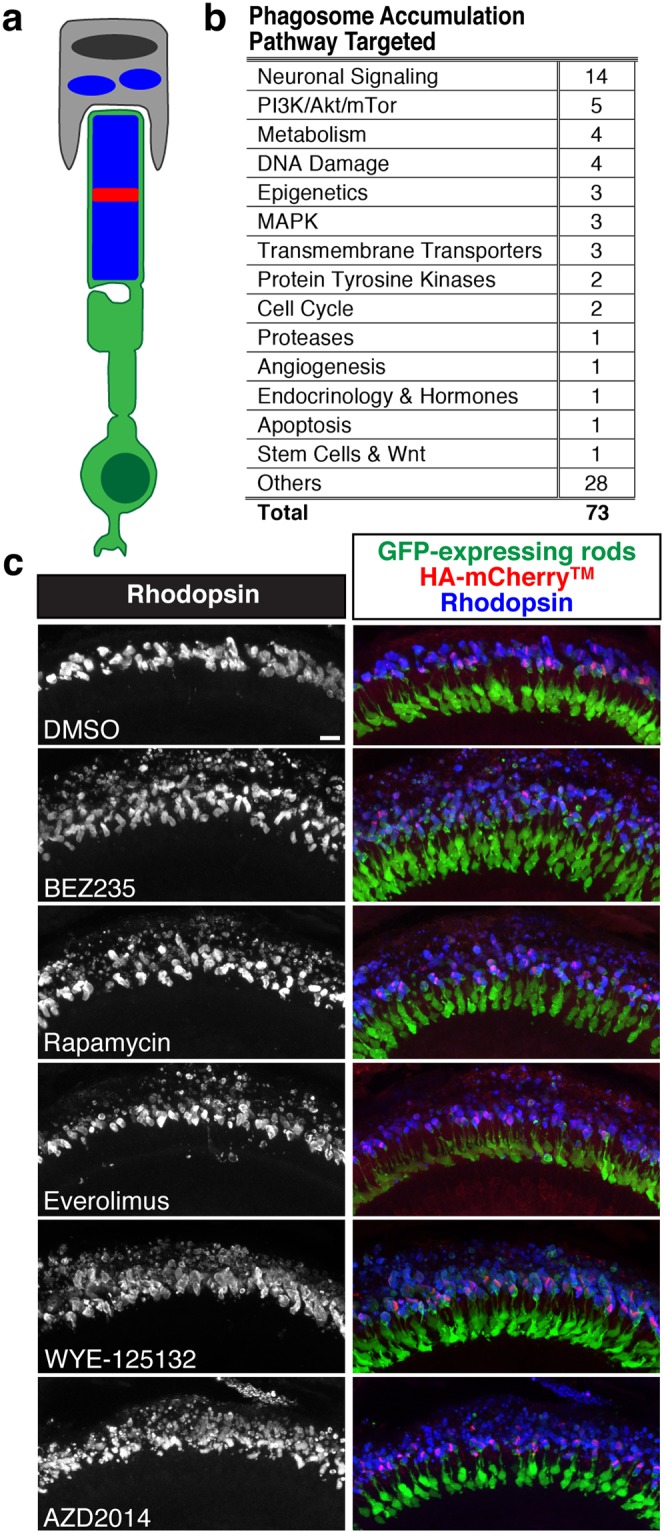
Phagosomes accumulate following treatment with PI3K/Akt/mTor inhibitors. (**a**) Accumulation of phagosomes, which are packets of shed ROS that can be detected with Rhodopsin immunolabeling, following disrupted RPE phagocytosis or digestion. (**b**) Signaling pathways targeted by compounds that resulted in phagosome accumulation. (**c**) Compounds that target the PI3K/Akt/mTor pathway lead to phagosome accumulation as compared to the DMSO control. Merged images show GFP-expressing rods in green, HA-mCherry^TM^ stripe in red, and Rhodopsin immunolabeling in blue. Scale bar is 10 μm.

Previous research has shown that ROS shedding occurs shortly after the onset of light when the phagocytic RPE recognizes, engulfs, and digests the distal tips of the ROS^4,9,26^. When RPE phagocytic function is disrupted, retinal health and function are compromised. For example, failure to engulf shed ROS material, as in animal models lacking MerTK or both of its ligands Gas6 and Protein S, leads to perturbed disc morphology, build-up of debris between the outer segments and RPE, and rapid retinal degeneration^16,19,27^. In addition, loss of the rhythmic increase in phagocytosis after light onset, as in mice lacking the αvβ5 integrin or ligand MFG-E8, causes accumulation of inclusion bodies and age-related vision loss^28,29^. Fluorescence microscopy with Rhodopsin immunolabeling has been used previously to identify Rhodopsin-positive phagosomes^29,30^, and so the image analysis-based design of this screen allowed us to identify compounds that led to an accumulation of Rhodopsin-positive phagosomes distal to the ROS (Fig. 4a).

We observed 73 compounds that resulted in phagosome accumulation. These compounds target pathways involved in neuronal signaling and mTor signaling (Fig. 4b). Many compounds that target the mTor pathway led to a striking increase in phagosomes (Fig. 4c). These compounds also decreased ROS growth (decreased D^G^) and increased shedding (decreased D^S^), suggesting that phagosome accumulation could be a consequence of increased ROS shedding, decreased RPE function, or both. Other compounds that increased phagosomes, including those that target MEK and adrenergic receptors did not increase ROS shedding, suggesting a direct effect on RPE function. The subset of data for compounds associated with phagosome accumulation is included in Supplementary Table S1.

### Bioactive compounds induce Rhodopsin mislocalization

**Figure 5:**
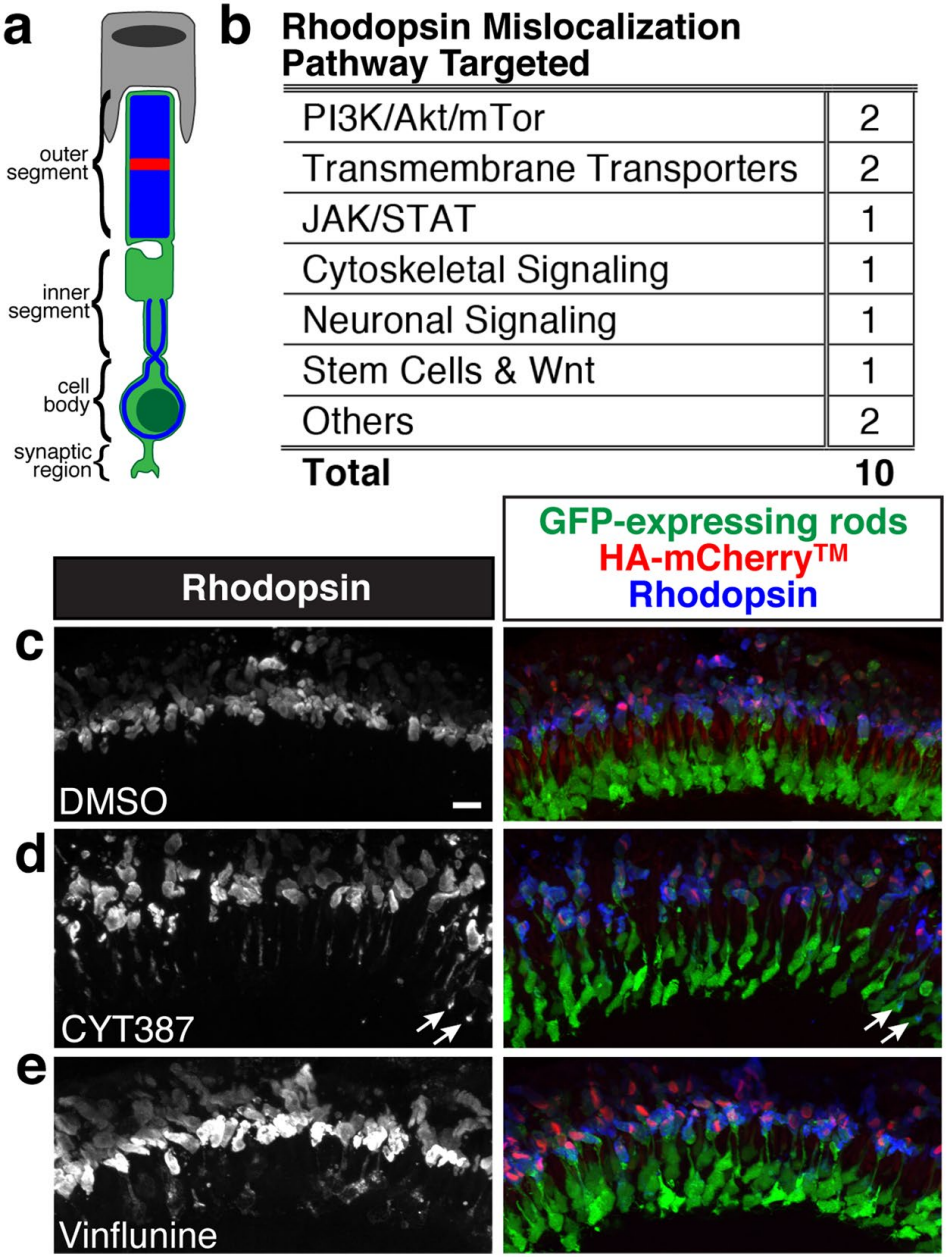
Rhodopsin mislocalizes following treatment with JAK1/2 and microtubule inhibitors. (**a**) Rhodopsin normally localizes to the ROS and is mislocalized when detected in the inner segment, cell body, or synaptic region with Rhodopsin immunolabeling. (**b**) Pathways targeted by compounds that lead to Rhodopsin mislocalization. (**c**) Rhodopsin localizes to the ROS in DMSO treated larva. (**d**) Rhodopsin is mislocalized to the inner segment, as well as some puncta in the cell body (arrows), following treatment with the JAK1/2 inhibitor CYT387. (**e**) Rhodopsin is mislocalized to the inner segment and cell body following treatment with Vinflunine, which disrupts microtubules. Merged images show GFP-expressing rods in green, HA-mCherry^TM^ stripe in red, and Rhodopsin immunolabeling in blue. Scale bar is 10 μm.

Rhodopsin mislocalization, which is characteristic of many forms of retinitis pigmentosa, is thought to be a major contributing factor in photoreceptor cell death^31,32^. The anti-Rhodopsin antibody used in this study normally labels Rhodopsin protein exclusively in the ROS. However, with some compounds we observed Rhodopsin mislocalization in the inner segment and cell body (Fig. 5a). We observed 10 compounds that caused Rhodopsin mislocalization, including compounds that target mTor signaling and transmembrane transporters (Fig. 5b). In the DMSO control, Rhodopsin was localized to the outer segments (Fig. 5c). CYT387, a JAK1/2 inhibitor^33^, led to Rhodopsin accumulation in the inner segment, as well as some Rhodopsin puncta in the cell body (Fig. 5d). Vinflunine, an inhibitor of microtubule assembly^34^, led to Rhodopsin mislocalization in both the inner segment and cell body (Fig. 5e). Some of the compounds that led to Rhodopsin mislocalization, including the two mTor signaling inhibitors AZ20 and TIC10, also resulted in decreased D^G^. The subset of data for compounds associated with Rhodopsin mislocalization is included in Supplementary Table S1.

### Bioactive compounds disrupt development of new rods from the CMZ

**Figure 6:**
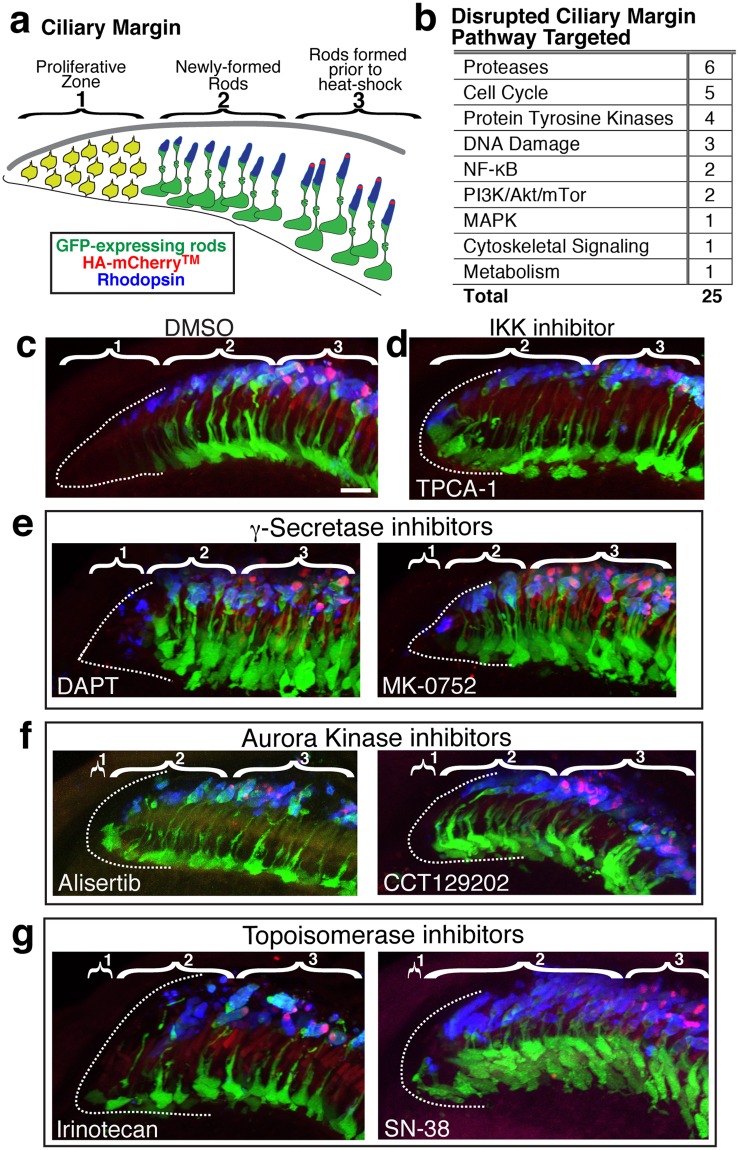
Notch signaling, cell cycle, and DNA damage pathways are among those targeted by compounds that disrupt the peripheral rim of the retina. (**a**) The peripheral rim of the retina consists of (1) a proliferative zone, the CMZ, where stem and precursor cells reside, (2) a region of newly-formed retinal neurons and glia including GFP-positive rods, and (3) rods that have the mCherry^TM^ stripe because they developed prior to heat shock. (**b**) Pathways targeted by compounds that disrupt the peripheral rim of the retina. (**c**) The peripheral rim from a control DMSO-treated larva segmented into region (1) with dotted white outline, (2) with GFP (green) and Rhodopsin (blue)-positive but mCherry^TM^ stripe-negative rods, and (3) with GFP, Rhodopsin, and mCherry^TM^ (red)-positive rods. (**d**) The proliferative zone (1) is nearly absent following treatment with IKK inhibitor TPCA-1. (**e**) The proliferative zone (1) is reduced in size and newly formed rods (2) are packed tightly with γ-Secretase inhibitor (DAPT and MK-0752) treatment. (**f**) The proliferative zone (1) is dramatically reduced with Aurora Kinase inhibitor (Alisertib and CCT129202) treatment. (**g**) The proliferative zone is dramatically reduced with topoisomerase inhibitor (Irinotecan and SN-38) treatment. Scale bar is 10 μm.

The CMZ is a stem cell niche at the periphery of the zebrafish retina that continues to add new cells throughout the life of the fish^35,36^. In the *Tg(hsp70:HA-mCherry*^*TM*^) retina, only rods that were formed prior to heat shock exhibit the mCherry^TM^ stripe. Therefore, we were able to qualitatively examine three regions of the peripheral rim of the retina: (1) the proliferative CMZ where there were no GFP-positive rods, (2) the region of newly formed rods that lacked the mCherry^TM^ stripe because they developed after heat shock and during compound treatment, and (3) the region of older rods with mCherry^TM^ stripes that developed before heat shock (Fig. 6a). We observed 25 compounds that affected the CMZ (Fig. 6b) as compared to DMSO control (Fig. 6c). Six of the compounds targeted proteases, all of which were γ-Secretase inhibitors. Cell cycle pathways, including four Aurora Kinase inhibitors, and protein tyrosine kinases were also targeted. Treatment with the IκB kinase 2 (IKK-2) inhibitor, TPCA-1 (categorized as pathway “NF-κB”), reduced the size of the proliferative zone and increased packing of new rods, which exhibited abnormal morphology (Fig. 6d). Treatment with γ-Secretase inhibitors, DAPT and MK-0752^37^, caused a reduction in the proliferative zone and crowding of the new rods (Fig. 6e). A nearly complete loss of the proliferative zone was observed with Aurora Kinase inhibitors, Alisertib and CCT129202^38,39^ (Fig. 6f). A similar loss of the proliferative zone was observed with the topoisomerase inhibitor Irinotecan and its active metabolite SN-38^40^ (Fig. 6g). The subset of data for compounds associated with disrupted CMZ is included in Supplementary Table S1.

## Discussion

Methodological limitations have delayed progress towards understanding the molecular mechanisms that regulate photoreceptor outer segment renewal. The recently developed transgenic *(hsp70:HA-mCherry*^*TM*^) zebrafish expresses a marker of ROS renewal, which permits independent analysis of growth and shedding^20^. The transgenic mCherry^TM^ stripe measurement tool combined with a small molecule screening approach provide a non-biased method to rapidly identify candidate molecular pathways. This approach has identified several candidate molecular pathways that regulate ROS renewal, as well as RPE phagocytosis, Rhodopsin mislocalization, and development of new rods at the CMZ. We were also attentive to potential effects on the integrity of the mCherry^TM^ stripe as an indication of abnormal disc formation but did not observe any such qualitative changes.

In order to screen as many compounds as possible, we limited the study to a single concentration over a single time course during larval ages. Additionally, given the workload of this screen, we were limited to processing a small number of fish for each compound (n = 3 unless otherwise noted in the Supplementary Table S1). However, the total number of ROS measured for each compound treatment was substantial and, in combination with two other factors in the analysis (1, setting a threshold for percent difference as compared to control, and 2, redundancy in molecular and pathway targets of the compounds tested), aided in identifying compounds of interest as modulators of ROS renewal. Thresholds for the increase and decrease of D^G^ and D^S^ were set at 20% difference as compared to the associated DMSO control. Benchmark values for D^G^ and D^S^ within this study were determined based on the analysis of all ROS measurements from DMSO control-treated fish (n = 213), while the threshold of 20% was somewhat arbitrarily selected. Nonetheless, with the complete analysis available in the Supplementary Table S1, the lists of compounds of interest can be adjusted based on higher or lower thresholds. Despite the threshold value, a benefit of the small molecule screen is the redundancy of molecules and pathways targeted by different compounds. The role of some targets, such as cyclooxygenases in ROS growth, γ-Secretases in ROS shedding, and mTor signaling in phagosome clearance, is strongly supported since multiple compounds that target the same protein or pathway exhibit similar effects on the rod photoreceptors. From the results of this screen, a subset of compounds that affect ROS renewal similarly, including those that do so beyond the threshold of 20% difference and those that do not hit the threshold but target the same molecule or pathway, can be retested with a variety of concentrations, with fish at different ages, and for different lengths of time. For example, we recently expanded a study on the effects of PDE 5/6 inhibitors on ROS shedding with dose response curves and in adult fish. One of those compounds, Vardenafil, increased D^S^ by 21%, while Sildenafil increased D^S^ by 14% as compared to control when tested at 20 μM within this screen. In extended study both Vardenafil and Sildenafil maximally increase D^S^ in larval and adult fish at the slightly higher concentration of 50 μM^14^.

Zebrafish are particularly amenable to small molecule screening in part because they can be bathed in water with a small amount of compound. Consequently, the entire animal is affected by the compound, and small molecule screening provides the added benefit of targeting both rod cell autonomous and non-cell autonomous pathways^41^. Although new membranous discs are added to the base of the ROS by rod cell autonomous protein synthesis and trafficking^15^, it is unclear how neighboring cells, such as Müller glia and RPE, contribute to the regulation of ROS growth. ROS shedding is, in part, regulated by phototransduction and ROS phosphodiesterase 6 activity^14^ but also requires non-cell autonomous regulation from the RPE^19,42^. Further experimentation will be required to determine which cell types are directly affected by each compound. Future approaches to study candidate pathways will need to rely on gene manipulations including knock-out and overexpression studies. The generation of conditional and inducible gene expression and knock-out systems, like the TetOn system, will aid in addressing the cell-specific roles of candidate genes^43^.

The distance between the base of the outer segment and the mCherry^TM^ stripe (D^G^) represents ROS growth that occurred during incubation with the compound. Several compounds that inhibit cyclooxygenases (COX), which are key regulators of inflammation, increased ROS growth. Although it has been shown that mouse rod photoreceptors express COX-2^44^, it remains to be determined whether the growth-promoting effect observed in this study is through direct inhibition of cyclooxygenases in rods. The RPE is also a potential target of these inhibitors as it has been shown that cultured rat RPE expresses *COX-1* constitutively while *COX-2* expression increases upon incubation with isolated ROSs^45^. Another pathway mediating inflammation, among other roles, is the angiotensin system, also known as the renin–angiotensin–aldosterone system (RAAS). We observed that several inhibitors of this pathway, specifically competitive antagonists of the angiotensin II type I (AT1) receptor^46^, also increased ROS growth. It does not appear that photoreceptors express AT1, at least not in rat^47^, however human and rat RPE do express AT1^47,48^.

Nearly three times as many compounds decreased ROS growth compared to those that increased growth. This was not surprising since we have observed that food deprivation during this larval period decreases ROS growth (AMJ, unpublished observation). Furthermore, the high energy consumption of photoreceptors for daily protein and membrane synthesis^49^ suggests that any compound that compromises the ability of the larva to ingest and metabolize their food or compromises overall larval growth will stunt ROS growth. We observed that many compounds affecting neuronal signaling pathways reduced ROS growth, predominantly those affecting serotonin signaling and glutamatergic signaling. These compounds may stunt ROS growth by altering feeding behavior or, in the case of serotonin signaling, disrupting gut function^50^. Similarly, compounds targeting the PI3K/Akt/mTor pathway, which also decreased ROS growth, may act systemically to reduce larval growth and secondarily reduce ROS growth. However as a central regulator of cell size^51^, these compounds may directly target rod cell growth by inhibiting the high metabolic rate these cells demand for daily maintenance.

ROS shedding distance (D^S^), measured as the distance between the mCherry^TM^ stripe and the ROS tip, represents growth that occurred before compound treatment minus any shedding that occurred during compound treatment. Therefore, a difference in D^S^ between DMSO-treated fish and compound-treated fish reflects a change in the rate of ROS shedding resulting from compound treatment. We observed few compounds that significantly suppressed shedding (i.e., increased D^S^). Four compounds that target cell cycle pathways, including inhibitors of CDK, Wee1, and Aurora Kinase, increased D^S^. Given that rods are post-mitotic, it seems unlikely that these compounds act directly as cell-cycle inhibitors on rods to increase D^S^. Although CCT129202 is a potent inhibitor of Aurora kinase^39^, it has also been shown to inhibit ABC transporters^52^. Whether CCT129202 inhibits the photoreceptor outer segment-specific ABC transporter, ABCA4^53^, remains to be determined. Compounds that target ubiquitous epigenetic pathways also increased D^S^, including two compounds that target HDACs and one that targets bromodomain and extraterminal (BET) proteins. Two closely related MEK5/ERK5 inhibitors increased D^S^, suggesting that MAPK pathway signaling regulates shedding. Reports of ERK5 expression in RPE suggest that MAPK signaling may regulate rod photoreceptor shedding non-cell autonomously^54,55^.

Nearly three times as many compounds increased ROS shedding (i.e., decreased D^S^) as compared to decreased shedding. The pathways most commonly targeted by compounds that increased shedding are similar to those that decreased ROS growth, including neuronal signaling, PI3K/Akt/mTor, and DNA damage. As discussed above with decreased ROS growth, the high energy demands of photoreceptors make them particularly susceptible to the compounds that compromise overall metabolism in the larva. Decrease in both D^G^ and D^S^ by these compounds suggests that maintenance of ROS length is metabolically demanding for the regulation of shedding as well as the maintenance of ROS growth. Several compounds that inhibit γ-Secretase increased shedding (decreased D^S^), and two of those (YO-01027 and LY11575) also decreased ROS growth. γ-Secretase expression in RPE maintains barrier function by mediating pigment epithelium-derived factor through processing of vascular endothelial growth factor^56,57^. In addition, several compounds that inhibit Aurora Kinase decreased D^S^, and inhibition of Aurora Kinase has been associated with ciliary axoneme stabilization in a RPE cell line^58^. Therefore, γ-Secretase and Aurora Kinase inhibitors acting locally within the retina may be regulating ROS shedding by disrupting RPE function.

Several compounds that target the PI3K/Akt/mTor pathway led to an increase in anti-Rhodopsin-labeled phagosomes as well as an increase in shedding (decreased D^S^). Increased shedding (decreased D^S^) is not always associated with increased phagosomes, suggesting that the phagocytic RPE is usually capable of clearing and digesting excess shed ROS material. Therefore, the increase in phagosomes with inhibition of the PI3K/Akt/mTor pathway may be due to direct effects on RPE function. Indeed, an analysis of RPE gene expression in the RCS rat (lacking *mertk* function) revealed early changes in the mTOR pathway^59^. Alternatively, several compounds that affect adrenergic signaling caused an increase in phagosomes (four α adrenergic receptor agonists and one α adrenergic receptor antagonist) without affecting D^S^. With α-adrenergic receptor expression reported in the RPE^60,61^, it is possible that these compounds act directly on RPE to modulate phagocytic activity. Based on our current understanding of RPE phagocytic activity, the compounds that result in phagosome accumulation could be (1) inhibiting complete engulfment of shed outer segments similar to the MerTK deficiency in the RCS rat^16,19,27^, (2) disrupting the synchronicity of phagocytosis after light onset as in mice lacking the αvβ5 integrin or ligand MFG-E8^28,29^, (3) delaying phagosome trafficking from the apical to the basal regions of RPE as in mice lacking the actin-associated proteins myosin VIIa or annexin A2^62,63^, or (4) delaying digestion of phagosomes as in mice lacking melanoregulin^64^.

Although many proteins and pathways have been identified that contribute to Rhodopsin trafficking and transport, such as small GTPases and the IFT complex^65,66^, we still lack a full understanding of the processes that regulate Rhodopsin trafficking and localization. In this study, relatively few compounds resulted in Rhodopsin mislocalization, which may be because the pathways that regulate Rhodopsin trafficking and localization are essential for larval viability^67^. Many of the compounds that did cause Rhodopsin mislocalization were associated with degenerating rods, and in most cases the mislocalization was observed in only some rods. Furthermore, we observed no Rhodopsin mislocalization with most of the compounds that caused ROS degeneration, suggesting that Rhodopsin mislocalization does not simply result from degeneration. This is consistent with observations that while Rhodopsin mislocalization has been observed in some mouse rod degeneration mutants (e. g., *rds*/*rom1*^68^ and *tulp1*^69^), Rhodopsin mislocalization is not associated with degeneration in other mutants (e. g., *mertk*^70^, *rpe65*, *lrat*^71^, and *cngb1*^72^).

Sustained growth of the zebrafish retina allowed us to screen for compounds that disrupted the proliferative zone and morphology of newly-formed rods at the CMZ. Most commonly, we identified compounds that target Notch signaling and cell cycle pathways, consistent with previous molecular characterization of the CMZ^35^. Both γ-Secretase and IKK/NF-kB are important components of Notch signaling^73^, and compounds that inhibit these proteins reduced the size of the proliferative zone and appeared to increase the number of newly formed rods, as reflected by abnormal rod density and disrupted morphology. Not surprisingly, compounds that target cell proliferation, either directly by targeting the cell cycle or indirectly by targeting DNA damage pathways, caused a qualitative reduction in the proliferative zone of the CMZ. A major limitation with this content area is the low *n* used to assess the CMZ. Despite this, the data are a useful report for identifying compounds and targets for further analysis.

A greater understanding of the molecular mechanisms that regulate ROS renewal will guide treatment and interventions for retinal degeneration diseases. Current hurdles in treating these diseases include a lack of druggable targets and crossing the blood-retinal barrier^74^. This screen assists in addressing the first hurdle of identifying candidate pathways as targets with the benefit that many of the compounds are FDA approved, which would expedite their use in therapeutic treatment. Furthermore, recent progress using a nanosized liposomal drug delivery system may facilitate the transport of potential therapeutic compounds across the blood-retinal barrier^74^. Prior to therapeutic intervention, however, further investigation with cell-autonomy centered experiments will be required to identify the cell types directly targeted by the compounds. To this end, we have created TetOn-based tools to manipulate gene expression in rod photoreceptors^43^ as well as in RPE (Jensen and Willoughby, unpublished). These cell-type specific TetOn tools and CRISPR/Cas9 approaches used along with the *Tg(hsp70:HA-mCherry*^*TM*^*)* tool provide the opportunity to make rapid progress in expanding our understanding of photoreceptor outer segment renewal and may illuminate therapeutic targets to slow or prevent the degeneration of outer segments, which is associated with, and possibly causal to, photoreceptor degeneration in disease.

## Materials and Methods

### Zebrafish care and maintenance

This study was carried out in strict accordance with the recommendations in the Guide for the Care and Use of Laboratory Animals of the National Institutes of Health. The protocol was approved by the University of Massachusetts Amherst Institutional Animal Care and Use Committee. All fish lines were maintained according to standard methods^75^ at 28 °C on a 14/10-hour light/dark cycle. All fish lines were a mixed *AB*/*albino*^*b4/b4*^ background^76,77^. The *Tg(Xla*.*rho:EGFP)* line was obtained from James Fadool (Florida State University, Tallahassee, FL, USA)^23^. The *Tg(hsp70:HA-mCherry*^*TM*^*)* line was described previously^20^.

### Pre-screen embryo and larva maintenance

Embryos were collected from mass matings, pooled together, and sorted into petri dishes at a density of 100 embryos per dish with egg water (0.3 g/L Instant Ocean Sea Salt; Spectrum Brands, Blacksburg, VA, USA). Embryos were maintained at 28–30 °C in a benchtop incubator until 6 dpf. At 5 dpf, fish were screened for GFP fluorescence in the retina and maintained in 5 ppt salt water (Instant Ocean Sea Salt; Spectrum Brands) with live rotifers at 1,000 rotifers/mL^78^.

### Heat shock and compound treatment

Transmembrane-associated mCherry (HA-mCherry^TM^) expression was induced at 6 dpf, 4 hours after the onset of light by incubation in 38 °C water for 45 minutes. After heat shock, fish were distributed into wells of 12-well tissue culture plates at 8 fish per well in 3 mL room temperature 5 ppt salt water (Instant Ocean Sea Salt; Spectrum Brands, Blacksburg, VA, USA) containing 20 μM bioactive compound (10 mM stock in DMSO, Bioactive Compound Library L1700, Selleck Chemicals, Houston, TX, USA) or 0.2% DMSO plus 1,000 rotifers/mL. Compounds were screened on a weekly basis at 8 to 32 compounds per week. In addition, DMSO vehicle control was processed each week so that compound-treated fish could be compared to control fish with as many environmental factors controlled for as possible (e. g., clutch, rotifer batch, temperature, water, etc.) Plates containing the control and compound-treated fish were maintained under standard fish facility conditions (28 °C on a 14/10-hour light/dark cycle) for 4 days and were examined daily. Water/compound was not exchanged. Dead fish were removed, and the number of rotifers remaining was monitored. At 1 day post heat shock, additional rotifers were not needed. Additional rotifers were added at 150–200 μL of 1,000 rotifers/mL on 2 days post heat shock to the DMSO control well and all other wells such that the number of rotifers present in each well, as monitored by eye, was similar to the DMSO control well levels. At 3 days post heat shock, rotifer numbers were similarly monitored and added as needed.

### Tissue Processing and Immunofluorescence

Fish were fixed at 10 dpf, 4 hours after the onset of light in 4% paraformaldehyde for 1 hr at room temperature. Fish were briefly washed in PBS and tails were removed. Heads were embedded in 1.5% agar/5% sucrose and equilibrated in 30% sucrose. Embedded tissues were sectioned at 30 μm thickness with a Leica cryostat. Tissue sections were rehydrated with PBS containing 0.1% Tween (PBS-Tw) for 15 minutes, blocked for 1 h with 20% goat serum in PBS-Tw, and incubated overnight at 4 °C with anti-GFP and anti-HA antibodies diluted with PBS-Tw. Sections were washed with PBS-Tw and incubated with secondary antibodies for 6 h at room temperature, washed with PBS-Tw, and incubated with anti-Rhodopsin antibody overnight at 4 °C. Sections were washed with PBS-Tw and incubated with secondary antibody for 6 h at room temperature. Slides were mounted with ProLong Gold Antifade Reagent (ThermoFisher Scientific, Waltham, MA, USA) after a final wash with PBS-Tw. Antibodies used include rabbit anti-GFP primary antibody at 1:1000 (ThermoFisher Scientific, Waltham, MA, USA) and corresponding Alexa Fluor 488-conjugated goat anti-rabbit secondary antibody at 1:1000 (ThermoFisher Scientific, Waltham, MA, USA), mouse IgG_1_ monoclonal anti-HA primary antibody at 1:1000 (Covance, Princeton, NJ, USA) and corresponding Rhodamine Red-conjugated goat anti-mouse IgG_1_ secondary antibody at 1:100 (Jackson ImmunoResearch Laboratories, West Grove, PA, USA), R6-5 mouse IgG_2A_ monoclonal anti-Rhodopsin primary antibody^79^ at 1:200 and corresponding Alexa Fluor 647-conjugated goat anti-mouse IgG_2A_ secondary antibody at 1:100 (Jackson ImmunoResearch Laboratories, West Grove, PA, USA).

### Image collection and analysis

Images were generated as z-stacks of optical sections using a Zeiss LSM 700 Confocal system with a 40 × /1.4 NA oil objective and processed with Zen software (Carl Zeiss, Thornhill, NY, USA) and Adobe Photoshop (San Jose, CA, USA). The photoreceptor layer from the central retina was imaged from three different individuals (unless otherwise noted in the Supplementary Table S1) and a single representative CMZ image was collected from one individual within the treatment group. Z-stacks were collected with a thickness of 24–28 μm with step size of 0.426 μm. Representative images are maximum intensity z-projections.

The Zeiss z-stack files (.lsm) were imported into Volocity 3D Image Analysis Software (PerkinElmer, Waltham, MA, USA), which accounts for distance between z-sections and allows for measurements to be made within the three-dimensional z-stack. Measurement analyses were performed using the line function by manually drawing from the base of the outer segment to the middle of the mCherry^TM^ stripe for D^G^ and from the middle of the mCherry^TM^ stripe to the tip of the outer segment for D^S^. The base and tip of the outer segments were determined by the Rhodopsin staining and morphology as indicated with the *Xop*:eGFP fluorescence. All ROS within an image were measured where the mCherry^TM^ stripe was present and the ROS base/tip were visible. Intensity of the fluorescence channels were adjusted with the Volocity software as needed per image to identify these landmarks. Confocal images were saved with coded file names according to week number and sample number such that the individuals who performed the measurement analysis did not know the compound identity.

For the qualitative content areas, z-stacks for the three outer segment layer images and the single CMZ image for each compound and control were converted to maximum intensity z-projections in the Zen Software (Carl Zeiss, Thornhill, NY, USA) and imported into Volocity 3D Image Analysis Software (PerkinElmer, Waltham, MA, USA). Each image for every compound and control was viewed and assessed in the far red channel for the presence of phagosome accumulation as determined by packets of positive anti-Rhodopsin immunofluorescence in the subretinal space distal to the ROS (Fig. 4). A compound of interest was determined if at least one of the three images showed accumulation of phagosomes. Similarly, the far red channel was assessed, independent of the green and red channels, for the presence of Rhodopsin immunofluorescence in rod cell bodies for the Rhodopsin mislocalization content area (Fig. 5). A compound of interest was determined if at least one of the three images showed Rhodopsin mislocalization. To identify the proliferative zone in the CMZ image (Fig. 6), brightness in the red channel was greatly increased in Adobe Photoshop (San Jose, CA, USA), the margin was traced with a dotted line, and then the red channel brightness was returned to original level.

### Statistics and quantification of outer segment renewal

Data were plotted using the ggplot2 package^80^ in R (version 3.2.3)^81^. Box plots represent the distribution of data with lower and upper hinges of the box corresponding to first and third quartiles and middle bar corresponding to the median. Whiskers extend 1.5 * interquartile range above and below the hinges; dots represent outliers. Large black dot represents the mean; small colored dots represent individual measurements with color corresponding to individual fish.

To analyze the D^G^ and D^S^ measurements, the data for each week of the screen were fit with mixed effects modeling using the lmerTest package in R^82^. Mean, standard deviation, and the total number of measured ROS for each compound and DMSO control were determined and compiled (plyr package^83^, R). D^G^ and D^S^ means for a compound were compared to the control D^G^ and D^S^ means for the DMSO control that was processed during the same week using Dunnett’s comparison with the adjusted *p*-values from the mixed effects modeling (multcomp package^84^, R). Significance levels are ***(*p* < 0.001), **(*p* < 0.01), and *(*p* < 0.05). Qualitative data were combined with measurement data, and then all data were sorted in Microsoft Excel (Redmond, WA, USA) according to content areas.

## Electronic supplementary material

Dataset 1

## Data Availability

The authors declare that the data supporting the findings of this study are available within the paper and its supplementary information files or can be obtained from the authors upon request.
